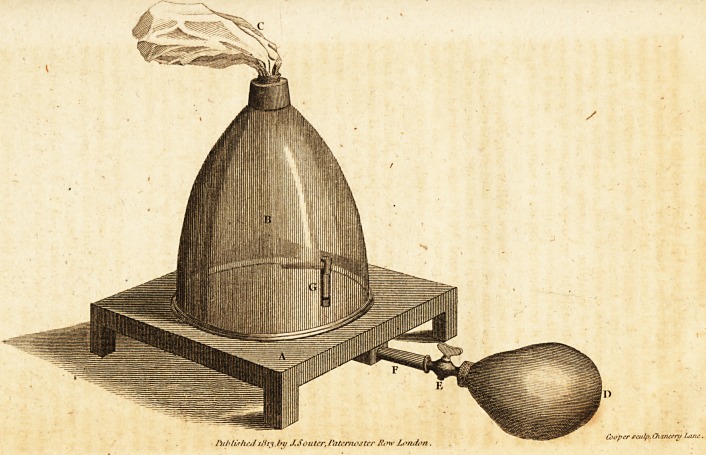# Collectanea Medica

**Published:** 1813-02

**Authors:** 


### COLLECTANEA MEDICA,

CONSISTING OF

### ANECDOTES, FACTS, EXTRACTS, ILLUSTRATIONS, QUERIES, SUGGESTIONS, &c.

RELATING TO THE

*History or the Art of Medicine, and the Auxiliary Sciences.*

---

*Further Experiments and Observations on the Influence of the Brain on the Generation of Animal Heat. By* B. C. BRODIE, Esq. F.R.S. *Read before the Royal Society, June* 18, 1812.

IN the Croonian Lecture for the year 1810, I gave an account of some experiments, which led me to conclude that the production of animal heat is very much under the influence of the nervous system. Some circumstances which I have since met with, illustrate this subject, and seem to confirm the truth of my former conclusions.

In an animal which is under the influence of a poison that operates by disturbing the functions of the brain, in proportion as the sensibility becomes impaired, so is the power of generating heat impaired also.

If an animal is apparently dead from a poison of this description, and the circulation of the blood is afterwards maintained by means of artificial respiration, the generation of heat is found to be as completely destroyed as if the head had been actually removed.

Under these circumstances, if the artificial respiration is kept up until the effects of the poison cease, as the animal recovers his sensibility, so does he also recover the power of generating heat; but it is not till the nervous energy is completely restored, that heat is produced in sufficient quantity to counteract the cold of the surrounding atmosphere.*

In the experiments formerly detailed, as well as in those just mentioned, I observed that the blood underwent the usual alteration of color in the two systems of capillary vessels, while carbonic acid was evolved from the lungs at each ex-

---

* The poison employed in this experiment should be the essential oil of almonds, or some other, the effects of which speedily subside. If the woorara is employed, so long a time elapses before the poison ceases to exert its influence, that it becomes necessary that the experiment should be made in a high temperature, otherwise the great loss of heat which takes place is sufficient to prevent recovery.

piration; piration; and hence I was led to believe that the respiratory function was performed nearly as under ordinary circumstances, and that the usual chemical changes were produced on the blood. It appeared, however, desirable to obtain some more accurate knowledge on this point, and I have, therefore, instituted a series of experiments for the purpose of ascertaining the relative quantities of air consumed in breathing, by animals in a natural state, and by animals in which the brain has ceased to perform its office; and I now have the honor of communicating an account of these experiments to this society.

It has been shewn, by Messrs. Allen and Pepys, first,* that every cubic inch of carbonic acid requires exactly a cubic inch of oxygen gas for its formation; secondly,† that, when respiration is performed by a warm-blooded animal in atmosperic air, the azote remains unaltered, and the carbonic acid exactly equals, volume for volume, the oxygen gas, which disappears.

There is, therefore, reason to believe, that the watery vapor which escapes with the air in expiration, is not formed from the union of hydrogen with oxygen in the lungs, but that it is exhaled from the mucous membrane of the mouth and pharynx, resembling the watery exhalation which takes place from the peritonæum, or any other internal surface, when exposed; and this conclusion appears to be fully confirmed by the experiments of M. Magendie, lately communicated to the National Institute of Paris.

These circumstances are of importance in the present communication, which they render more simple, as they shew that, in order to ascertain the changes produced on the air in respiration, it is only necessary to find the quantity of carbonic acid given out from the lungs. This becomes an exact measure of the oxygen consumed, and the azote of the air and the watery vapor expired, need not be taken into the account.

For the purpose of examining the changes produced on the air, by animals breathing under the different circumstances above-mentioned, I contrived the apparatus, which is represented in the annexed Plate.

*Description of the Apparatus.*—A is a wooden stand, in which is a circular groove three quarters of an inch in depth, and the same in width.

B is a bell-glass, the rim of which is received in the circular groove

* Phil. Trans. 1807. Part II.

† Phil. Trans. 1808. Part II. Ibid. 1809. Part II.

Published 1813, by J. Souter, Paternoster Row London.

Cooper sculp. Chancery Lane.

groove of the wooden stand. In the upper part of the bell-glass is an opening, admitting a tube connected with the bladder C.

D is a bottle of elastic gum, having a brass stop-cock E connected with it.

F is a silver tube, of which one end is adapted to receive the tube of the stop-cock E, while the other extremity, making a right angle with the rest of the tube, passes through a hole in the wooden stand, and projects into the cavity of the bell-glass, where it makes a second turn also at a right angle, and becomes of a smaller diameter. In the upright part of the tube is an opening G.

The tubes are made perfectly air-tight, where connected with each other, and with the rest of the apparatus; and the circular groove is filled with quicksilver.

The capacity of the bell-glass, allowance being made for the rim, which is received in the groove with the quicksilver, is found to be 502 cubic inches. The capacity of the gum-bottle is 52 cubic inches, and, in the calculations after the experiments, two cubic inches have been allowed for the air contained in the different tubes, and for the small remains of air in the bladder after being nearly emptied by pressure.

*Mode of using the Apparatus.*—In order to ascertain the quantity of air consumed under ordinary circumstances, the animal was placed on the stand under the bell-glass, the bladder being emptied by pressure, and the gum-bottle being distended with atmospheric air. During the experiment, by pressing occasionally on the gum-bottle, the air was forced from it into the bell-glass. On removing the pressure, the gum-bottle became filled by its own elasticity with air from the bell-glass. Thus the air was kept in a state of agitation, and the dilatation of the bladder prevented the air being forced through the quicksilver under the edge of the bell-glass. At the end of the experiment, the gum-bottle was completely emptied by pressure, and allowed to be again filled with air from the bell-glass: this was repeated two or three times, and the air in the bottle was then preserved for examination. The proportion of carbonic acid being ascertained, and the capacities of the different parts of the apparatus, and the space occupied by the animal, being known, the total quantity of carbonic acid formed, and consequently of oxygen consumed, was easily estimated.

When the experiment was made on an animal in whom the functions of the brain were destroyed, and in whom, therefore, voluntary respiration had ceased, the narrow extremity of of the tube was inserted into an artificial opening in the trachea, and, the animal being placed under the bell-glass, the lungs were inflated at regular intervals, by means of pressure made on the gum-bottle. The tube being smaller than the trachea, the greater portion of the air in expiration escaped by the side of the tube into the general cavity of the bell-glass, while the gum-bottle filled itself by its own elasticity with air through the opening G. At the end of the experiment, a portion of air was preserved for examination, and the quantity of carbonic acid was estimated in the way already described.

The animals employed in these experiments were of the same species, and nearly of the same size. Attention to these circumstances was judged necessary, that the results might be as conclusive as possible. The chemical examination of the air was made by agitating it in a graduated measure over quicksilver, with a watery solution of potash. My friend, Mr. Brande, gave me his assistance in this part of the present investigation, as he had done on many former occasions. It will be observed, that in estimating the proportion of carbonic acid, no allowance has been made for that contained in the atmospheric air; first, because the quantity is so small that the omission can occasion no material error; and secondly, because the object is to ascertain, not so much the absolute, as the relative, quantities of carbonic acid evolved by animals breathing under different circumstances.

The experiments which I shall first notice, were made on the respiration of animals in a natural state.

*Experiment* 1. Thermometer 65°, barometer not noted.

A young rabbit was allowed to remain under the bell-glass during 30 minutes. The respired air at the end of this time was found to contain $\frac{1}{20}$ of carbonic acid.

It was ascertained that the rabbit occupied the space of 50 cubic inches.

The capacity of the bell-glass = 502 cubic inches.

That of the gum-bottle 52 cubic inches.

The air in the tubes and bladder = 2 cubic inches.

$$\text{Then } \frac{502 + 52 + 2 - 50}{20} = \frac{506}{20} = 25.3.$$

The rabbit therefore in 30 minutes gave out 25.3 cubic inches of carbonic acid, and consumed the same quantity of oxygen gas, which is at the rate of 50.6 in an hour.

*Exp.* 2. Thermometer 65°, barometer 30.1 inches.

A somewhat smaller rabbit was allowed to remain under the bell-glass during 30 minutes. The respired air contained tained $\frac{1}{18}$ of carbonic acid. The animal occupied the space of 48 cubic inches.

$$\frac{502 + 52 + 2 - 48}{18} = \frac{508}{18} = 28.22.$$

The carbonic acid evolved was therefore equal to 28.22 cubic inches in half an hour, which is at the rate of 56.44 cubic inches in an hour.

*Exp.* 3. Thermometer 64°, barometer 30.2 inches.

A young rabbit, occupying the space of 48 cubic inches, was allowed to remain under the bell-glass, during the same period as in the two former instances. The respired air contained $\frac{1}{18}$ of carbonic acid.

$$\frac{502 + 52 + 2 - 48}{18} = \frac{508}{18} = 28.22.$$

The results of this were therefore precisely the same as those of the last experiment.

These experiments were made with great care. The animals did not appear to suffer any inconvenience from their confinement, and their temperature was unaltered.

The next order of experiments were made for the purpose of ascertaining the quantity of air consumed by animals, in which the circulation of the blood was kept up by means of artificial respiration, after the brain had ceased to perform its functions.

*Exp.* 4. Thermometer 65°, barometer not noted.

Having procured two rabbits of the same size and color, I divided the spinal marrow in the upper part of the neck of one of them. An opening was made in the trachea, and the lungs were inflated at first by means of a small pair of bellows. Two ligatures were passed round the neck, one in the upper, and the other in the lower, part, behind the trachea. The ligatures were drawn tight, including every thing but the trachea; and the nerves, vessels, and other soft parts between them were divided with a bistoury. Eight minutes after the division of the spinal marrow, the thermometer in the rectum had sunk to 97°. The animal was placed under a bell-glass, and the lungs were inflated by pressing on the gum-bottle about 50 times in a minute. When this process had been continued for 30 minutes, a portion of air was preserved for examination. The heart was found acting regularly, but slowly; the thermometer in the rectum had fallen to 90°.

The second rabbit was killed by dividing the spinal marrow about the same time when the experiment was begun on the first rabbit. Being in the same temperature, the time was noted when the thermometer in the rectum had fallen to 97°, and it was placed under another bell-glass, that it might be as nearly as possible under the same circumstances with the first rabbit. At the end of 30 minutes, the thermometer in the rectum had fallen from 97 to 91.*

The air respired by the first rabbit contained $\frac{1}{25}$ of carbonic acid. The bulk of the rabbit was found = 50 cubic inches.

$$\frac{502 + 52 + 2 - 50}{25} = \frac{506}{25} = 20.24.$$

20.24 cubic inches of carbonic acid were therefore extricated in 30 minutes, which is at the rate of 40.48 cubic inches in an hour.

The carbonic acid given out in the same space of time was less than in the former experiments; but it is to be observed, first, that in consequence of the ligatures the extent of the circulation was diminished; secondly, that in this instance one of the ligatures accidentally slipped, and an ounce of blood was lost in the beginning of the experiment.

As it was desirable to avoid any circumstances which might occasion a difference in the results, in the subsequent experiments, I employed animals which had been inoculated with the poison of woorara, or the essential oil of almonds; by which means, while the functions of the brain were completely destroyed, the extent of the circulation was undiminished, and all chance of accidental hæmorrhage was avoided.

*Exp.* 5. Thermometer 65°, barometer 29.8 inches.

Two rabbits were procured, each occupying the space of 45 cubic inches. They were both inoculated with the woorara poison.

The first rabbit was apparently dead in nine minutes after the application of the poison; but the heart continued to act. The lungs were inflated for about two minutes, by means of a pair of bellows, when the thermometer in the rectum was observed to stand at 98°. The animal was placed under the bell-glass, and artificial respiration was produced by means of pressure on the gum-bottle, as in the last experiment. At the end of 30 minutes, a portion of air was preserved for examination. The thermometer in the rectum had fallen to 91°. The heart still acted with regularity and strength.

* In measuring the heat of the rectum in these experiments, care is necessary that the thermometer should always be introduced to exactly the same distance from the external parts, otherwise no positive conclusion can be drawn relative to the loss of heat, as the more internal parts retain their heat longer than the superficial.

The

The second rabbit died in a few minutes after the inoculation. The time was noted when the thermometer in the rectum had fallen to 98°, and he was placed under a bell-glass. At the end of 30 minutes, the thermometer in the rectum had fallen to 92°.

The air respired by the first rabbit contained $\frac{1}{20}$ of carbonic acid.

$$\frac{502 + 52 + 2 - 45}{20} = \frac{511}{20} = 25.55$$ cubic inches of carbonic acid evolved in 30 minutes, which is at the rate of 51.1 cubic inches in an hour.

*Exp.* 6. Thermometer 66°, barometer 30.1 inches.

Two rabbits, each occupying the space of 48 cubic inches, were inoculated with woorara.

In one of them, when apparently dead, the circulation was kept up by means of artificial respiration. He was placed in the apparatus under the bell-glass, and the lungs were inflated from 50 to 60 times in a minute. At this time the thermometer in the rectum stood at 97°. At the end of 35 minutes, a portion of air was preserved for examination. The thermometer had now fallen to 90°. The heart was still acting regularly.

The second rabbit was allowed to lie dead. When the thermometer in the rectum had fallen to 97°, he was placed under another bell-glass. At the end of 35 minutes, the thermometer had fallen to 90°.5.

The air respired by the first rabbit contained $\frac{1}{16}$ of carbonic acid.

$$\frac{502 + 2 + 52 - 48}{16} = \frac{508}{16} = 31.75$$ cubic inches of carbonic acid evolved in 35 minutes, which is at the rate of 54.43 cubic inches in an hour.

*Exp.* 7. Thermometer 60°, barometer 30.2 inches.

The experiment was repeated on a rabbit, which had been inoculated with the essential oil of almonds. When he was placed under the bell-glass, the thermometer in the rectum stood at 96°. In a few minutes he gave signs of sensibility, and made efforts to breathe; but, as these efforts were at long intervals, the artificial respiration was continued. In half an hour he breathed spontaneously 40 times in a minute. The thermometer in the rectum had fallen to 90°.

The air, being examined, was found to contain $\frac{1}{18}$ of carbonic acid.

The rabbit occupied the space of 47 cubic inches.

$$\frac{502 + 52 + 2 - 47}{18} = \frac{509}{18} = 28.275$$ cubic inches of carbonic acid evolved in 30 minutes, which is at the rate of 56.55 cubic inches in an hour.

The animal lay as if in a state of profound sleep. At the end of two hours and twenty minutes, from the time of the poison being applied, the thermometer in the rectum had fallen to 79°, and he was again apparently dead; but the heart still continued acting, though feebly, and its action was kept up for 30 minutes longer by means of artificial breathing, when the thermometer had fallen to 76°. The carbonic acid evolved during these last 30 minutes, amounted to nearly 13 cubic inches.

From the precautions with which these experiments were made, I am induced to hope that there can be no material error in their results. They appear to warrant the conclusion, that, in an animal in which the brain had ceased to exercise its functions, although respiration continues to be performed, and the circulation of the blood is kept up to the natural standard, although the usual changes in the sensible qualities of the blood take place in the two capillary systems, and the same quantity of carbonic acid is formed as under ordinary circumstances; no heat is generated, and (in consequence of the cold air thrown into the lungs) the animal cools more rapidly than one which is actually dead.

It is a circumstance deserving of notice, that so large a quantity of air should be consumed by the blood passing through the lungs, when the functions of the brain, and those of the organs dependant on it, are suspended. Perhaps it is not unreasonable to suppose, that, by pursuing this line of investigation we may be enabled to arrive at some more precise knowledge respecting the nature of respiration, and the purposes which it answers in the animal economy. It would, however, be foreign to the plan of the present communication to enter into any speculations on this subject, and I shall therefore only remark, that the influence of the nervous system does not appear to be necessary to the production of the chemical changes, which the blood undergoes in consequence of exposure to the air in the lungs.*

The

---

* This conclusion is directly contrary to that deduced by M. Dupuytren, from a series of curious experiments, made with a view to ascertain the effects which follow the division of the nerves of the par vagum, and it is an object of some importance in the present investigation, to ascertain in what manner the apparently opposite facts, observed by M. Dupuytren and myself, are to be reconciled with each other.

It was observed by this physiologist, that in an animal, in which both the nerves of the par vagum are divided, the blood returned from the lungs has a darker color than natural, and that the animals, on whom

The facts now, as well as those formerly, adduced, go far towards proving that the temperature of warm-blooded animals is considerably under the influence of the nervous system; but what is the nature of the connection between them? whether is the brain directly or indirectly necessary to the production of heat? These are questions to which no answers can be given, except such as are purely hypothetical. At present we must be content with the knowledge of the insulated fact: future observations may, perhaps, enable us to refer it to some more general principle.

We have evidence that when the brain ceases to exercise its functions, although those of the heart and lungs continue to be performed, the animal loses the power of generating heat. It would, however, be absurd to argue from this fact, that the chemical changes of the blood in the lungs are in no way necessary to the production of heat, since we know of no instance in which it continues to take place after respiration has ceased.

It must be owned that this part of physiology still presents an ample field for investigation.

Of opinions sanctioned by the names of Black, Laplace, Lavoisier, and Crawford, it is proper to speak with caution

---

whom this operation is performed, die sooner or later with symptoms of asphyxia, notwithstanding the air continues to enter the lungs; and hence he concludes, that the changes which are produced on the blood in respiration are not the result of a mere mechanical process, but are dependent on the nervous influence, and cease to take place when the communication between the lungs and the brain is destroyed.

M. Provençal, in prosecuting this inquiry, ascertained that the animals subjected to this experiment give out less carbonic acid than before.

M. Blainville observed, that the frequency of the inspirations is much diminished; and M. Dumas restored the scarlet color of the arterial blood by artificially inflating the lungs; and, from these and other circumstances, he has arrived at conclusions very different from those of M. Dupuytren.

My own observations exactly correspond with those of MM. Dumas and Blainville. After the nerves of the par vagum are divided, a less quantity of carbonic acid is evolved, the inspirations are much diminished in frequency, and the blood in the arteries of the general system assumes a darker hue; but its natural color may be restored by artificially inflating the lungs, so as to furnish a greater supply of air to the blood circulating through them.

We may suppose, that, on the division of these nerves, the sensibility of the lungs is either extremely impaired, or altogether destroyed, so that the animal does not feel the same desire to draw in fresh air: in consequence, his inspirations become less frequent than natural, and hence arise the phenomena produced by this operation.

and and respect; but, without trespassing on these feelings, I may be allowed to say, that it does not appear to me that any of the theories hitherto proposed, afford a very satisfactory explanation of the source of animal heat.

Where so many and such various chemical processes are going on, as in the living body, are we justified in selecting any one of these for the purpose of explaining the production of heat?

To the original theory of Dr. Black, there is this unanswerable objection, that the temperature of the lungs is not greater than that of the rest of the system. To this objection, the ingenious and beautiful theory of Dr. Crawford is not open; but still it is founded on the same basis with that of Dr. Black, "the conversion of oxygen into carbonic acid in the lungs," and hence it appears to be difficult to reconcile either of them with the results of the experiments which have been related.

It may, perhaps, be urged, that, as in these experiments the secretions had nearly, if not entirely, ceased, it is probable that the other changes which take place in the capillary vessels had ceased also; and that, although the action of the air on the blood might have been the same as under ordinary circumstances, there might not have been the same alteration in the specific heat of this fluid, as it flowed from the arteries into the veins. But, on this supposition, if the theory of Dr. Crawford be admitted as correct, there must have been a gradual but enormous accumulation of latent heat in the blood, which we cannot suppose to have taken place without its nature having been entirely altered. If the blood undergoes the usual change in the capillary system of the pulmonary, it is probable that it must undergo the usual change in the capillary system of the greater circulation also, since these changes are obviously dependent on, and connected with, each other. The blood in the aorta and pulmonary veins was not more florid, and that in the vena cava and pulmonary artery was not less dark-colored, than under ordinary circumstances. We may, moreover, remark, that the most copious secretions in the whole body, are those of the insensible perspiration from the skin, and of the watery vapor from the mouth and fauces; and the effect of these must be to lower, rather than to raise, the animal temperature. Under other circumstances also the diminution of the secretions is not observed to be attended with a diminution of heat. On the contrary, in the hot fit of a fever, when the scanty dark-colored urine, dry skin, and parched mouth, indicate that scarcely any secretions are taking place, the the temperature of the body is raised above the natural standard, to which it falls when the constitution returns to its natural state, and the secretions are restored.

It has been observed, by a distinguished chemist, that "the experiments to determine the specific heat of the blood are of so very delicate a nature, that it is difficult to receive them with perfect confidence."* The experiments of Dr. Crawford for this purpose were necessarily made on blood out of the body, and at rest. Now, when blood is taken from the vessels, it immediately undergoes a remarkable chemical change, separating into a solid and a fluid part. This separation is not complete for some time; but, whoever takes the pains to make observations on the subject, can hardly doubt that it begins to take place immediately on the blood being drawn. Can experiments on the blood, under these circumstances, lead to any very satisfactory conclusions, respecting the specific heat of blood circulating in the vessels of the body? The diluting the blood with large quantities of water, as proposed by Dr. Crawford, does not altogether remove the objection, for this only retards, it does not prevent, coagulation, and some time must, at any rate, elapse, while the blood is flowing and the quantity is being measured, during which the separation of its solid and fluid parts will have begun to take place.

More might be said on this subject; but I feel anxious to avoid, as much as possible, controversial discussion. It is my wish, not to advance opinions, but simply to state some facts which I have met with in the course of my physiological investigations. These facts, I am willing to hope, possess some value; and they may, perhaps, lead to the developement of other facts of much greater importance. Physiology is yet in its infant state. It embraces a great number and variety of phenomena; and of these it is very difficult to obtain an accurate and satisfactory knowledge; but it is not unreasonable to expect, that, by the successive labors of individuals, and the faithful register of their observations, it may at last be enabled to assume the form of a more perfect science.—*Phil. Trans.*

---

* Thomson's History of the Royal Society, p. 129.

CRITICAL

## Figures and Tables

**Figure f1:**